# Ganoderma lucidum Triterpenoids (GLTs) Reduce Neuronal Apoptosis via Inhibition of ROCK Signal Pathway in APP/PS1 Transgenic Alzheimer's Disease Mice

**DOI:** 10.1155/2020/9894037

**Published:** 2020-01-28

**Authors:** Nanhui Yu, Yongpan Huang, Yu Jiang, Lianhong Zou, Xiehong Liu, Sulai Liu, Fang Chen, Jun Luo, Yimin Zhu

**Affiliations:** ^1^First Affiliated Hospital of Hunan Normal University (Hunan Provincial People's Hospital), Changsha, Hunan 410005, China; ^2^Hunan Provincial Institute of Emergency Medicine, Hunan Provincial Key Laboratory of Emergency and Critical Care Metabonomic, Changsha, Hunan 410005, China; ^3^Changsha Social Work College, Changsha, Hunan 410004, China; ^4^Department of Hepatobiliary Surgery, Hunan Provincial People's Hospital, Changsha, Hunan 410005, China; ^5^Hunan Research Center of Biliary Disease, Hunan Provincial People's Hospital, Changsha Hunan 410005, China; ^6^Guizhou Medical University, Guiyang, Guizhou 550025, China; ^7^Health Commission of Hunan Province, Changsha, Hunan 410013, China

## Abstract

Alzheimer's disease (AD) is the most common cause of dementia among senior citizen. Ganoderma lucidum triterpenoids (GLTs) have nutritional health benefits and has been shown to promote health and longevity, but a protective effect of GLTs on AD damage has not yet been reported. The objective of this research was to elucidate the phylactic effect of GLTs on AD model mice and cells and to explore its underlying mechanisms. Morris water maze (MWM) test was conducted to detect changes in the cognitive function of mice. Hematoxylin-eosin (HE) staining was applied to observe pathological changes in the hippocampus. Silver nitrate staining was applied to observe the hippocampal neuronal tangles (NFTs). Apoptosis of the hippocampal neurons in mouse brain tissue was determined by TUNEL staining. The expression levels of apoptosis-related protein Bcl2, Bax, and caspase 3/cleaved caspase 3; antioxidative protein Nrf2, NQO1, and HO1; and ROCK signaling pathway-associated proteins ROCK2 and ROCK1 were measured by western blot. *In vivo* experiments show that 5-month-old APP/PS1 mice appeared to have impaired acquisition of spatial learning and GLTs could reduce cognitive impairment in AD mice. Compared to normal mice, the hippocampus of APP/PS1 mouse's brains was severely damaged, while GLTs could alleviate this symptom by inhibiting apoptosis, relieving oxidative damage, and inactivating the ROCK signaling pathway. In *in vitro* cell experiments, A*β*_25-35_ was applied to induce hippocampal neurons into AD model cells. GLTs promoted cell proliferation, facilitated superoxide dismutase (SOD) expression, and inhibited malondialdehyde (MDA) and lactic dehydrogenase (LDH) expression of neurons. Our study highlights that GLTs improve cognitive impairment, alleviate neuronal damage, and inhibit apoptosis in the hippocampus tissues and cells in AD through inhibiting the ROCK signaling pathway.

## 1. Introduction

Alzheimer's disease (AD), a widespread, progressive, nonreversible, and devastating set of neurodegenerative disorders, is characterized by progressive impairment of memory, motion disorders, and judging and reasoning abilities that eventually results in aphronesia [1, 2]. The pathological features mainly include senile plaques (SP) formed by extracellular amyloid *β*-protein (A*β*) deposition and endocellular neurofibrillary tangles (NFTs) formed by hyperphosphorylation of tau protein in neurons, along with the increasingly attenuate number and capacity of synapses and neurons [3]. The NFTs are composed of amyloid fibrils, which are associated with synapse loss and neurodegeneration, and eventually lead to memory impairment and other cognitive problems [4]. The pathogenic causes linked with the incidence of AD include poor mental performance, traumatic brain injury, cerebral stroke, low social activity, age, social exclusion, and physical inactivity, and low education level [5]. There is an estimated 46.8 million people worldwide who suffered from AD or interrelated dementia disease in 2015, and the morbidity of AD throughout the world is anticipated to exceed 1.315 billion by year 2050 [6, 7]. The disease is clinically basically characterized by a severe dysfunction of cognition and ascensive degradation of memory, resulting in loss of self-care ability and eventually needing all-time medical solicitude [8]. Nevertheless, up to now, there appears no efficient cure or prevention for AD, and the treatments simply moderate symptoms without affecting the disease development, laying a tremendous millstone on public health and society.

*Ganoderma lucidum* (*G*. *lucidum*) is a Basidiomycetes fungus from the order Polyporales and acclaimed officinal agaric used as a folk remedy in Asia since ancient times as a result of its multitudinous health-promoting capabilities [9, 10]. It has been manifested that this eumycete is beneficial in preventing and treating high blood pressure, hyperglycemia, chronic bronchitis, hepatitis, asthma, cancer, heart diseases, and HIV [11–13]. *Ganoderma lucidum* triterpenoids (GLTs) is the major variety of bioactive and medicative components in *Ganoderma lucidum* (*G*. *lucidum*) [14]. The mother nucleus made of isoprene is an important chemical structure of GLTs [15]. But the chemical structures of GLTs are more sophisticated as a result of the highly enriched oxidized states of these compounds. Most GLTs exhibit a large scale of bioactivity, including anticancer [16], antihypertensive, anti-HIV-1, antiangiogenic, immunomodulatory [17], antiandrogenic, antioxidant, antihepatitis B, antimicrobial activities, and anticomplement [18]. GLTs are efficient as adjuvant therapies and enhance health when united with other pharmaceuticals to cure fatigue syndrome, hepatitis, and prostate cancer [19]. However, research investigating the mechanism and application of *G*. *lucidum* or GLTs in the treatment of diseases remains preliminary in terms of both the utilization efficacy and product type. Moreover, the therapeutic effect and molecular mechanism of GLTs on AD needs further research.

The present research was designed to evaluate the possible neuroprotective impact of GLTs on antiapoptosis in AD course. Firstly, we determined the effect of GLTs on cognitive disorder in APP/PS1 transgenic AD model mice compared to the control normal mice by place navigation test and spatial probe test. Furthermore, in consideration of the neurons, apoptosis is an important pathological process in AD; the roles of GLTs in the hippocampus' apoptosis of mice were investigated. What's more, we also probed into the function of GLTs on A*β*_25-35_-induced hippocampal neuron cell AD model. In addition, it was found that ROCK signal pathway may participate in the regulation process of GLTs on AD. We hope that improved mechanistic understanding of these phenomena may lay the foundation for selecting new drugs and screening targets to treat this devastating disease.

## 2. Materials and Methods

### 2.1. Animal

Male APPswe/PS1dE9 (APP/PS1) transgenic mice with a C57BL/6J background and nontransgenic littermates (C57BL/6 mice) were purchased from the Jiangsu ALF Biotechnology Co., Ltd (Nanjing, China). All mice were housed under controlled room temperature (20–24°C) and humidity (60–80%) and received food and water ad libitum. Three-month-old APP/PS1 mice and C57BL/6 mice were used for this study, and the experimental protocol was approved by the Medicine Animal Welfare Committee of the First Affiliated Hospital of Hunan Normal University (Hunan Provincial People's Hospital).

### 2.2. Drug Treatment

GLTs were provided by the Supercritical Fluid Technology Research Center, Institute of Geochemistry, Chinese Academy of Sciences (Guiyang, China). Donepezil was purchased from Apharm Co., Ltd. (Daegu, South Korea). A total of 50 APP/PS1 mice were separated into five experimental groups: AD group (10 mL kg^−1^ normal saline), low-dose GLT group (0.35 g kg^−1^ GLTs), high-dose GLT group (1.40 g kg^−1^ GLTs), positive control group (0.38 g kg^−1^ donepezil), and solvent group (10 mL kg^−1^ edible oil). The above dosage was given by gavage once a day for 60 days. The other 10 C57BL/6 mice were in the normal control group (10 mL kg^−1^ normal saline).

### 2.3. Morris Water Maze (MWM) Test

An MWM test (recording system produced by Techman, Chengdu, China) was conducted after the intragastric gavage of GLTs and donepezil to determine changes in cognitive ability. The MWM experiments were divided into place navigation test and spatial probe test. The mice in place navigation test were trained once a day in the morning lasting for 9 days. The mice were put into the water from any of the three quadrants outside the safe platform to the wall of the pool, and the time of finding the safe platform within 120 s (escape latency) and the length of the swimming path (search distance) were recorded, and the mice were allowed to stay on the platform for 10 s. If the security platform cannot be found after 120 s, it is recorded as 120 s, and the mice were guided to the security platform. The place navigation test's results, including the escape latency and search distance, were represented as the average of the results obtained in 1-9 days. The safety platform was removed on the 10th day to conduct spatial probe test. The mice were put into the water from the same entry point, and the search time (exploration time) of the mice in the quadrant of the original safety platform within 120 s was recorded, as well as the percentage of the swimming distance of the rats in the quadrant of the original safety platform in the total distance (exploration distance percentage). The experiments were carried out at 8 am and 3 pm to preclude the influence of circadian rhythm, and the laboratory was kept quiet, and the temperature and light intensity were as consistent as possible.

### 2.4. Hematoxylin-Eosin (HE) Staining

The morphological changes of the CA1 area of the mouse hippocampus was observed by pathological examination. At the end of the MWM experiment, the mice were cut and their brain tissue was removed and quickly placed on the ice tray along the sagittal suture. The brain tissues were fixed in 4% formalin solution at 4°C for 8 h, taken out in a 70% ethanol solution for 5 min, then placed in 80%, 90%, 95%, and absolute ethanol for gradient dehydration for 4 h each time, respectively; finally, tissues were immersed in xylene for 30 min, and then embedded in paraffin. Continuous coronal sections at the optic chiasma area (including the hippocampus) were taken, and the slice thickness was 3 *μ*m. Each specimen was taken for 10 consecutive slices for the HE tests.

### 2.5. Silver Staining

Silver nitrate staining was applied to observe neuronal tangles (NFTs) in the CA1 area of the mouse hippocampus. In brief, the paraffin section was dewaxed and placed in a 20% silver nitrate aqueous solution and immersed in a 37°C incubator for 30 min in the dark; after distilled water washing for 3 min, 10% of the formaldehyde solution was treated for several seconds until the section was yellow; after washing with distilled water for 5 min, the ammonia silver droplets were applied to dye for 40 s; 10% formaldehyde was again treated for 2 min and then 5% sodium thiosulfate solution was used to fix for 5 min. Finally, NFTs were observed under an optical microscope.

### 2.6. TUNEL Staining

Apoptosis of neurons in the CA1 area of the mouse hippocampus was measured by TUNEL staining (Roche, Nutley, NJ, USA). The paraffin-embedded tissue was cut into 4-5 *μ*m thick sections. The sections were then incubated in 50 *μ*L of the TUNEL mixture (47.5 *μ*L of TUNEL label containing fluorescein isothiocyanate-conjugated dUTP and 2.5 *μ*L of TUNEL enzyme) in a humidified chamber (60 min, 37°C). Control sections were incubated with 50 *μ*L of TUNEL label solution containing no TUNEL enzyme. Sections were photographed and TUNEL-positive nuclei were detected with IP Lab Imaging Analysis Software (Fairfax, VA, USA). Apoptotic index was calculated using TUNEL-positive nuclei/total number of nuclei×100 automatically.

### 2.7. Western Blotting Analysis

RIPA lysate (Beyotime, Shanghai, China) was used to obtain total proteins, 100 *μ*g of which were segregated using SDS-polyacrylamide gel electrophoresis and transferred onto polyvinylidene difluoride (PVDF) membranes. TBST containing 5% skim milk was used for membrane incubation for 1 h. Then, the membranes experienced incubation with primary antibodies including Anti-Bax antibody (ab77566, 1 *μ*g/mL, Abcam, Cambridge, MA, USA), anti-Bcl-2 antibody (ab196495, 1 : 500, Abcam), anti-Caspase-3 antibody (ab13847, 1 : 500, Abcam), anti-Cleaved Caspase-3 antibody (ab2302, 1 *μ*g/mL, Abcam), anti-Nrf2 antibody (ab137550, 1 : 500, Abcam), anti-Heme Oxygenase 1 antibody (ab13243, 1 : 2000, Abcam), anti-NQO1 antibody (ab2346, 0.3 *μ*g/mL, Abcam), anti-ROCK1 antibody (EP786Y) (ab45171, 1 : 2000, Abcam), anti-ROCK2 antibody (ab71598, 1 *μ*g/mL, Abcam), and anti-GAPDH (ab181603, 1 : 10000, Abcam) at 4°C overnight. The membranes were washed in TBST three times and incubated with anti-rabbit IgG H&L (HRP) secondary antibody (ab6721, 1 : 2000, Abcam) at room temperature for 1.5 h. After washing using TBST thrice, the membranes were subjected to color reaction by ECL Plus from Life Technology, and GAPDH was detected as control groups.

### 2.8. Primary Culture of Hippocampal Neurons

C57BL/6 mice born for 0-48 h were taken out and the brain was taken aseptically after sacrifice. The hippocampus tissue was isolated, washed in a Petri dish containing D-Hank's solution, and then 1-2 mL 0.02% EDTA and 0.25% trypsin digest were added into the dish; the hippocampus tissues were cut into small pieces, then transferred into a 10 mL centrifuge tube for digesting for 10-15 min; then, 4-6 mL DMEM/F12 medium containing 20% fetal bovine serum to terminate digestion was added to the decomposition liquor; the digestive solution was filtered with nylon sieve, centrifuged at 1000 rpm for 10 min, and cells were collected. Hippocampus cells were seeded in a 96-well culture plate at 1 × 10^6^ cells at 200 *μ*L per well and incubated in a 37°C, 5% CO_2_ incubator. After 7 days of culture, cells were used for follow-up experiments.

### 2.9. AD Cell Model and Drug Administration

Amyloid *β*-protein 25-35 (A*β*_25-35_, Sigma-Aldrich, St. Louis, MO, USA) was applied to induce hippocampal neuron cells into AD cells. At first, A*β*_25-35_ was diluted to a concentration of 5 *μ*g/*μ*L with sterile physiological saline and incubated at 37°C for one week to become aggregated A*β*_25-35_. Hippocampal neuron cells were divided into six groups: blank control group (control), model group (A*β*_25-35_), low-dose GLT group (3.0 *μ*mol L^−1^ GLTs), middle-dose GLT group (30.0 *μ*mol L^−1^ GLTs), high-dose GLT group (300.0 *μ*mol L^−1^ GLTs), and vehicle group (drug control group which added an equivalent medium and 20 *μ*mol L^−1^ A*β*_25-35_). Hippocampal nerve cells in the treatment group were added with GLTs at the above dosage, and the model group was added with the same amount of culture medium. After 24 h of culture, A*β*_25-35_ with a concentration of 20 *μ*mol L^−1^ was added to both the treatment group and the model group, and the culture was continued for 24 h. The blank control group was given the same amount of medium.

### 2.10. MTT Assay

Hippocampus cells were inoculated into the 96-well plates at a density of 1 × 10^4^ cells/well and stored in DMEM with FBS in it. At 37°C, cells were incubated for 4 h and 50 *μ*L of MTT (0.5 mg/mL, Sigma-Aldrich, St. Louis, MO, USA) was added into each well and had their growth condition observed at the 12th h. To solubilize the crystals, 150 *μ*L dimethylsulfoxide (DMSO) was added into each well after the supernatant was subsequently removed. The optical density (OD) was measured at 590 nm by a microplate reader (Bio-Rad, Hercules, CA, USA).

### 2.11. Determination of SOD, MDA, and LDH Levels

After drug treatment, the levels of superoxide dismutase (SOD), malondialdehyde (MDA), and lactic dehydrogenase (LDH) in the hippocampal neuron cell were then measured using a SOD test kit, MDA test kit, and LDH test kit, which were purchased from Nanjing Jiangcheng Bioengineering Institute (Nanjing, China). The experiment was conducted in accordance with the instructions of the kit. The parallel experiment was repeated three times.

### 2.12. Flow Cytometry Analysis

To detect cell apoptosis, PE Annexin V (BD Biosciences, San Jose, CA, USA) was applied, keeping to the instructions of the manufacturers. An FACSCalibur FCM (BD Biosciences) was used to observe cell apoptosis. Experiments in triplicate helped to reduce errors. FACS Diva software was adopted at data analysis.

### 2.13. Statistical Analysis

Experimental data analyses are performed with GraphPad Prism v6.0 statistical software and presented as mean ± standard deviation (SD) of results from three or more independent repetition experiments. Student's *t*-test was used to compare the differences between two groups. One-way ANOVA was applied to analyze three groups or above. *P* values < 0.05 accepted as the scale for statistical significance.

## 3. Results

### 3.1. Ganoderma lucidum Triterpenoids (GLTs) Alleviate Cognitive Impairment of AD Mice

**Figure 1:**
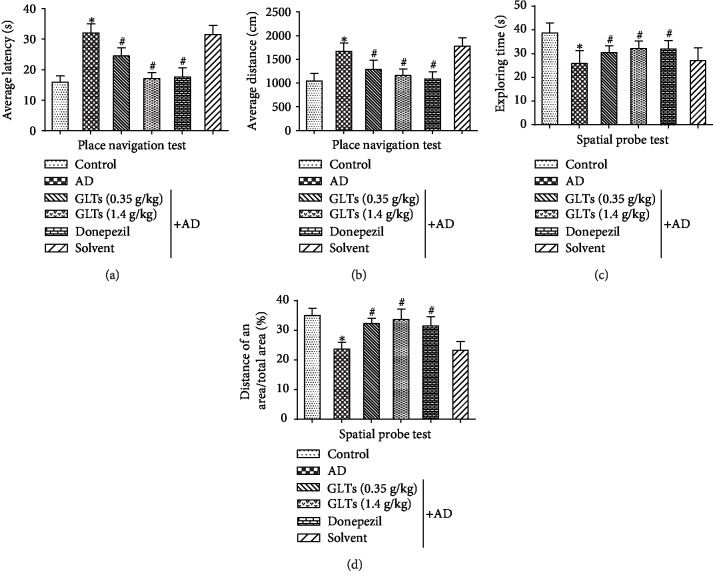
GLTs alleviate cognitive impairment of APP/PS1 transgenic AD mice. (a) The average escape latency of place navigation test in 1-9 days in different groups. (b) The average search distance of place navigation test in 1-9 days in different groups. (c) The space exploration time of spatial probe test in different groups. (d) The exploration distance percentage of spatial probe test in different groups. *N* = 6; ^∗^*P* < 0.05 compared to the control group, ^#^*P* < 0.05 compared to the AD group.

To explore the effect of GLTs on the 3 month APP/PS1 mouse's symptoms of dementia in behavior, it was detected with Morris water maze (MWM) test. The MWM test in our study included place navigation test and spatial probe test to analyze and infer the learning and memory, spatial orientation, and cognitive level of AD mice. As the results of place navigation test showed in Figures 1(a) and 1(b) , compared to the normal C57BL/6 mice, the average escape latency of occult platform (Figure 1(a)) and average search distance (Figure 1(b)) increased in AD mice. But the average escape latency period was shortened and the average search distance was reduced after gavage of GLTs. The results of the spatial probe test revealed that GLTs increased space exploration time (Figure 1(c)) and exploration distance percentage (Figure 1(d)), alleviating cognitive impairment of AD mice. These results suggest that the 5-month-old APP/PS1 mice presented impaired acquisition of spatial learning and GLTs could reduce cognitive impairment in AD mice.

### 3.2. Effects of GLTs on the Hippocampal Tissue Structure and Neuronal Tangles in the Hippocampus of AD Mice

**Figure 2:**
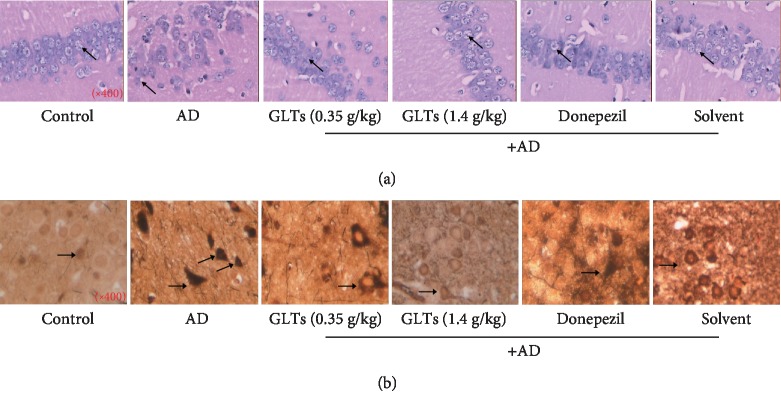
Effects of GLTs on the hippocampal tissue structure and neuronal tangles of the hippocampus of APP/PS1 transgenic mice. (a) HE staining was applied to present pathological changes in hippocampus tissues of mice in each group (×400). (b) The number of neuronal tangles (NFTs) in the CA1 area of the mouse hippocampus was detected by silver staining in each group (×400), *N* = 6.

In order to more intuitively reflect the effect of GLTs on the improvement of the hippocampal tissue of AD mice, HE staining was applied and presented in Figure 2(a). In the normal control group, the nerve cells were arranged neatly and round; the cell structure was intact and the cell membrane and nucleus were clear; and there was no obvious swelling and necrosis. The nerve cells in the AD group were extremely disordered and irregular in size and shape. The number of nerve cells was dramatically reduced, and the cell structure was blurred. After gavage of GLA in AD mice, high integrity of nerve cells was maintained and there was no obvious necrosis of nerve cells (Figure 2(a)). As neurofibrillary tangles (NFT) are an important pathological feature of AD patients, the number of NFT in the cytoplasm was detected. As showed in Figure 2(b), in the AD model mice, the number of neurons in the cortex and hippocampus decreased and the number of NFT greatly increased. Compared to the model group mice, the number of neurons in the cortex and hippocampus increased and the number of NFT in the cytoplasm significantly decreased in the GLT group.

### 3.3. Effects of GLTs on Apoptosis and Oxidative Damage in the Hippocampal Area of AD Mice through ROCK Signaling Pathway

**Figure 3:**
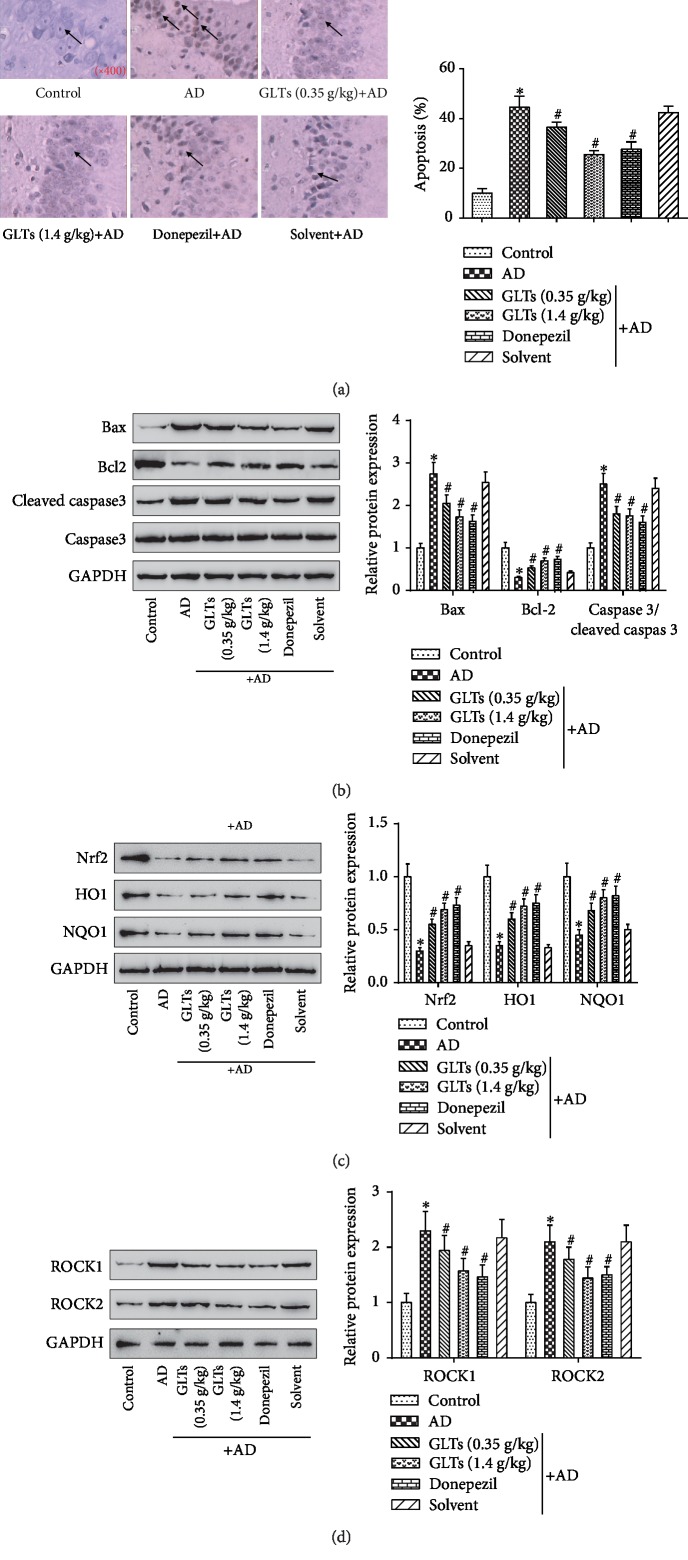
Effects of GLTs on apoptosis and oxidative damage in hippocampal area of APP/PS1 transgenic mice through ROCK signaling pathway. (a) The number of apoptotic positive cells in the CA1 area of the mouse hippocampus was measured in each group by TUNEL assay (×400). (b) The expression of apoptosis-related protein Bax, Bcl2, and caspase 3/cleaved caspase 3 in each group was detected by western blot. (c) Western blot analysis of the antioxidative proteins Nrf2, HO1, and NQO1 expression levels in the mouse hippocampus. (d) The protein expression of ROCK signaling pathway-associated proteins ROCK1 and ROCK2 in hippocampal tissues was determined. *N* = 6; ^∗^*P* < 0.05 compared to the control group, ^#^*P* < 0.05 compared to the AD group.

To explore the mechanisms by which GLTs improve cognitive impairment in AD mice, apoptosis, oxidative damage, and ROCK signaling pathways were investigated. As TUNEL assay, compared to the normal control group, the number of apoptotic positive cells in the AD group observably increased, but the number of apoptotic positive cells in the GLT group and the positive drug group prominently decreased (Figure 3(a), *P* < 0.05). Subsequently, the expression levels of apoptosis-related protein Bax, Bcl2, and caspase 3/cleaved caspase 3 in different groups were detected. The Bax and caspase 3/cleaved caspase 3 protein expression were dramatically elevated while Bcl2 protein expression decreased in the AD group. However, GLTs could reduce the apoptosis of neurons. Compared to the AD group, GLTs markedly reduced Bax and caspase 3/cleaved caspase 3 protein expression and increased Bcl2 protein (Figure 3(b), *P* < 0.05). Then, the expressions of antioxidative protein Nrf2 and the downstream antioxidant enzymes NQO1 and HO1 were detected in the AD mouse hippocampus (Figure 3(c)). The Nrf2, NQO1, and HO1 protein levels were notably declined in the AD group, while GLTs recovered the antioxidative protein levels in the AD mice. As displayed in Figure 3(d), the protein expression of ROCK1 and ROCK2 in the hippocampal tissues were dramatically enhanced in the AD group, but GLTs attenuated the increase of ROCK signaling pathway-associated proteins in AD mice (*P* < 0.05). It leads to the conclusion that GLTs inhibit apoptosis, relieve oxidative damage, and inactivate the ROCK signaling pathway to play a protective role in AD mice.

### 3.4. The Protective Effect of GLTs on Hippocampal Neuron AD Model Cells

**Figure 4:**
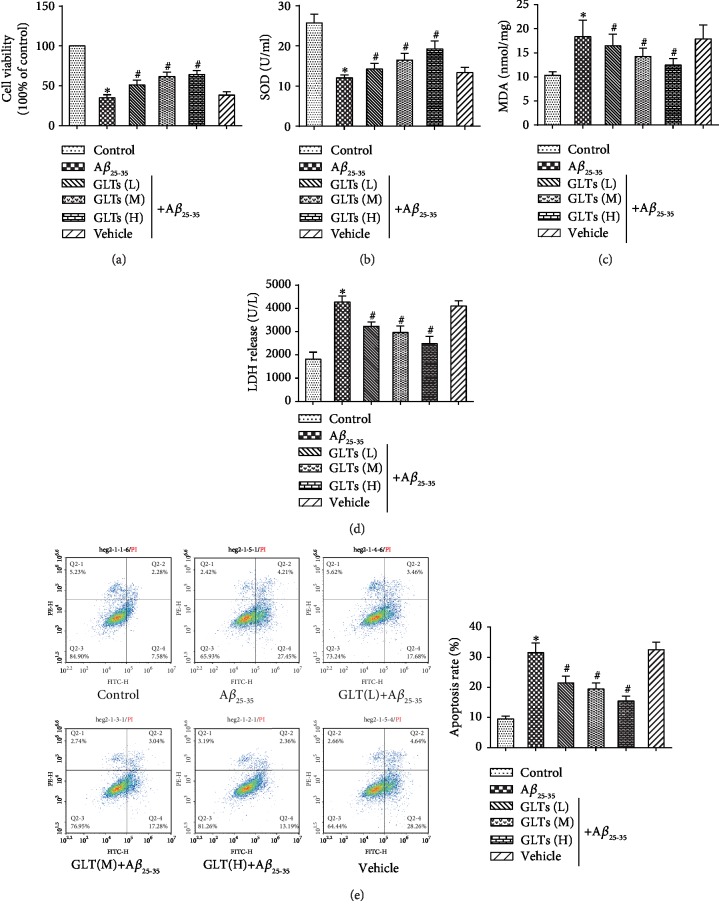
The protective effect of GLTs on hippocampal neuron AD model cells. Hippocampal neuron AD model cells were induced by A*β*_25-35_ treatment. (a) Cell proliferation activity of neurons under A*β*_25-35_ or GLT treatment was determined by MTT assay. (b) The SOD expression level in each group was measured by SOD test kit. (c) The MDA expression level in each group was measured by MDA test kit. (d) The LDH expression level in each group was measured by LDH test kit. (e) The apoptosis rate in each group was detected by flow cytometry analysis. *N* = 3; ^∗^*P* < 0.05 compared to the control group, ^#^*P* < 0.05 compared to the A*β*_25-35_ group.

To figure out the protective effect of GLTs on AD *in vitro*, AD model cells were established by A*β*_25-35_ treatment. Cell proliferation activity of neurons under A*β*_25-35_ or GLT treatment was determined by MTT assay. Compared to normal neurons, A*β*_25-35_ notably inhibited cell viability, while GLTs alleviated the inhibitory effect of A*β*_25-35_ on neuron proliferation (Figure 4(a), *P* < 0.05). To explore the antioxidant effects of GLTs, the expression level of SOD, MDA, and LDH were measured. As presented in Figure 4(b), the SOD expression level was observably declined in A*β*_25-35_-treated neurons (*P* < 0.05). Conversely, different concentrations of GLTs could facilitate SOD expression. However, the MDA (Figure 4(c)) and LDH (Figure 4(d)) expressions in the A*β*_25-35_ group were markedly aggrandized compared to the control group (*P* < 0.05). GLTs decreased the MDA and LDH levels to protect the hippocampal neurons. Then, the effect of different concentrations of GLTs on neurons' apoptosis was detected (Figure 4(e)). The apoptosis rate was prominently fortified in the A*β*_25-35_ group, while GLT treatment could restrain apoptosis of the hippocampal neurons (*P* < 0.05). The results suggested that GLTs play an antioxidant and inhibit apoptosis of the hippocampal neurons.

### 3.5. GLT Protects Hippocampal Neurons by Inhibiting the ROCK Signaling Pathway

**Figure 5:**
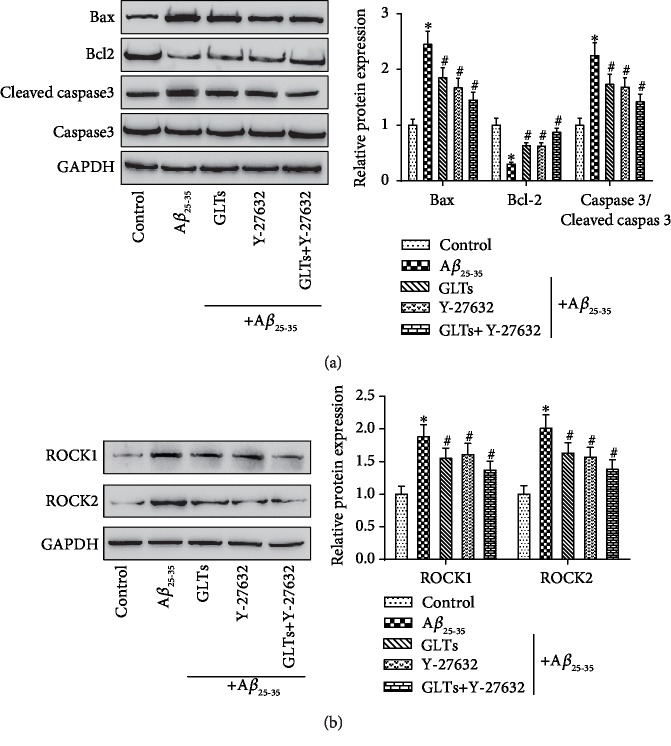
GLTs protect the hippocampal neurons from A*β*_25-35_ damage by inhibiting the ROCK signaling pathway. The ROCK signaling pathway inhibitor Y-27632 was added in the process of experiment. (a) The expression of apoptosis-related protein Bax, Bcl2, and caspase 3/cleaved caspase 3 in each group was detected by western blot. (b) The protein expression of ROCK signaling pathway-associated proteins ROCK1 and ROCK2 in each group was determined. *N* = 3; ^∗^*P* < 0.05 compared to the control group, ^#^*P* < 0.05 compared to A*β*_25-35_ group.

In order to explore whether GLTs protect hippocampal neurons by inhibiting ROCK signaling pathway and apoptosis, the ROCK signaling pathway inhibitor Y-27632 was added in the process of the experiment. The results in Figure 5(a) revealed that A*β*_25-35_ remarkably promoted Bax and caspase 3/cleaved caspase 3 protein expression and inhibited Bcl2 protein expression. While GLT or Y-27632 treatment memorably restrained Bax and caspase 3/cleaved caspase 3 protein expression and facilitated Bcl2 protein expression (*P* < 0.05). Next, the ROCK1 and ROCK2 proteins were detected by western blot (Figure 5(b)). A*β*_25-35_ prominently facilitates ROCK1 and ROCK2 protein expressions compared to normal neurons (*P* < 0.05). The inhibitory effect of GLTs on ROCK1 and ROCK2 proteins was consistent with that of Y-27632. Collectively, these data indicated that GLTs inhibit apoptosis and deactivate the ROCK signaling pathway to protect the hippocampal neurons from AD.

## 4. Discussion

Alzheimer's disease (AD) is a progressive neurodegenerative disease characterized by continuous cognitive decline and worsening of daily living performance, with no effective treatment as yet, and lays a tremendous burden on human society [20, 21]. As the pathogenic mechanism of AD is intricate and multifaceted, there is currently no effective therapeutic drugs to prevent or treat AD. Two types of drugs, acetyl choline esterase (AChE) inhibitor and N-methyl D-aspartate (NMDA) receptor antagonist, have been clinically prescribed for patients to improve their symptoms, yet without halting or reversing the pathological process [22, 23]. For centuries, many medicinal herbs and dietary supplements have been applied to ameliorate cognitive function and relieve symptoms associated with AD [24, 25]. *G*. *lucidum*, an edible medicinal mushroom, has diverse bioactivities including antidiabetes, antitumor, and immunomodulation and has been proved to ameliorate health and longevity for centuries in the Orient [26]. Huang et al. demonstrated that the polysaccharides from *G*. *lucidum* could ameliorate cognitive function and neural progenitor proliferation in a mouse model of AD [27]. However, as the major variety of bioactive and medicative components of *G*. *lucidum*, the therapeutic effect and molecular mechanism of GLTs on AD are not clear yet. In the present research, the APP/PS1 transgenic mouse model *in vivo* and A*β*_25-35_-induced hippocampal neuron cell model *in vitro* of AD had been established to investigate the effect of GLTs on the therapeutic action of AD. Animal experiments showed that 5-month-old APP/PS1 mice revealed impaired acquisition of spatial learning and GLTs could reduce cognitive impairment in AD mice. Besides, the pathological features of AD include the presence of amyloid plaques, neuronal death, and neurofibrillary tangles (NFTs) [28]. We also found that the number of NFT in the cytoplasm in AD mice significantly decreased after GLT treatment. Subsequent cell experiments also showed that GLTs alleviated the inhibitory effect of A*β*_25-35_ on neuron proliferation and had antioxidant effects. Here, we evidence that GLTs ameliorated cognitive dysfunction in transgenic AD model mice.

More recently, apoptosis has been attached great concern as an essential determinant in the pathological course of AD [29]. Apoptosis is a sequence of programmed events leading to the activation of caspases and cell disintegration [30]. Apoptosis plays crucial roles in tissue homeostasis, and squint towards being upregulated in neurodegenerative disease [31]. In AD, the processes have been proverbially studied but the contribution to neuronal death remains unclear. In our study, the effect of GLTs on apoptosis of hippocampal tissues and neurons in AD mice was studied in detail. GLTs decreased apoptosis rate and the expression of apoptosis-related protein Bax, Bcl2, and caspase 3/cleaved caspase 3 in both hippocampal tissues and neuron cells to play a protective role in AD mice.

What's more, the ROCK signaling pathway had also been found to be involved in AD progression. We found the protein expression of ROCK1 and ROCK2 in hippocampal tissues and neuron cells were dramatically enhanced in AD mice, but GLTs attenuated the increase of ROCK signaling pathway-associated proteins in AD mice. Consistent with our findings, Park et al. identified that RhoA-ROCK signaling pathway was activated in A*β*_42_-induced blood–brain barrier (BBB) [32]. Moreover, as a pivotal adjuster of cytoskeletal proteins, ROCK1/ROCK2 activity is considerable for the structural maintenance of neuronal processes which underlie synaptic transmission and cognitive functions [33]. These findings, along with our research results, indicate that ROCK signaling is the most determinate risk factor of AD [34]. In addition, oxidative stress damage in the hippocampus of the brain is the crucial pathologic changes during the early phase of AD [35]. Patients with slight cognitive impairment had been identified to have oxidative damage before evolving into AD [36]. Our results disclosed that antioxidative protein Nrf2, HO1, and NQO1 in the hippocampus of transgenic AD model mice was observably reduced, and GLT treatment counteracted this attenuation. This result indicated that GLTs could potentially exert a protective effect on AD. Similar to our results, Özevren et al. provide evidence for *G*. *lucidum* protects rat brain tissue against trauma-induced oxidative stress [37]. So, we concluded that GLTs relieved oxidative damage and inactivated the ROCK signaling pathway to play a protective role in AD mice and the multitarget effects of GLTs may have desirable advantages for the treatment of multifactorial neurodegenerative diseases such as AD.

However, the limitations of this research have yet to be considered. Despite progress in revealing therapeutic potentials of *G*. *lucidum* or GLTs, the complete regulation molecular targets of GLTs require additional research disclosure. Besides, the application of GLTs to the treatment of human diseases, including AD, requires more rigorous and scientific verification and clinical trials.

In conclusion, we discovered that GLTs relieved cognitive impairment and decreased NFT numbers in APP/PS1 transgenic AD model mice by inhibiting apoptosis and inactivating the ROCK signaling pathway. Besides, we revealed that GLTs facilitated hippocampal neuron proliferation and played antioxidant effect *in vitro* experiments. These findings may provide new clues for future therapeutic target research for this deadly disease.

## Data Availability

The data used to support the findings of this study are included within the article.
